# Endometriosis at the Single-Cell Level: Molecular Insights and Implications for Assisted Reproduction Success

**DOI:** 10.3390/biom16030402

**Published:** 2026-03-09

**Authors:** Angeliki Gerede, Efthymios Oikonomou, Foteini Gkaitatzi, Maria Danavasi, Panayiota Papasozomenou, Anastasios Potiris, Sofoklis Stavros, Vasiliki Kourti, Aikaterini Domali, Nikoletta Koutlaki, Menelaos Zafrakas

**Affiliations:** 1 Department of Obstetrics and Gynecology, Democritus University of Thrace, 691 00 Campus, Greece; efthymiosoikonomou@gmail.com (E.O.); gaitatzi.foteini@4ype-pfy.gr (F.G.); mairidanavasi@gmail.com (M.D.); vkourti@auth.gr (V.K.); nkoutlak@med.duth.gr (N.K.); 2 School of Health Science, International Hellenic University, 574 00 Thessaloniki, Greece; ppapasoz@ihu.gr; 3 Department of Obstetrics and Gynecology, University General Hospital “ATTIKON”, Medical School, National and Kapodistrian University of Athens, 124 62 Athens, Greece; apotiris@med.uoa.gr (A.P.); sfstavrou@med.uoa.gr (S.S.); 4 Department of Obstetrics and Gynecology, Alexandra Hospital, Medical School, National and Kapodistrian University of Athens, 115 28 Athens, Greece; kdomali@yahoo.fr

**Keywords:** endometriosis, assisted reproduction, assisted reproductive technologies (ART), single-cell analysis, molecular mechanisms, cellular heterogeneity, infertility, oocyte quality, endometrial receptivity

## Abstract

Endometriosis is a chronic hormone-responsive disorder linked to infertility, usually characterized by the presence of ectopic endometrium in the pelvis that disrupts local homeostasis. Advances in single-cell “omic” methods have revealed the remarkable cellular diversity within the eutopic endometrium and endometriosis lesions, uncovering distinct populations with unique transcriptional and functional profiles. These studies have highlighted alterations in immune cell subsets, stromal and epithelial cell signaling, and intercellular communication networks that collectively impair oocyte quality, embryo development, and endometrial receptivity in women with endometriosis. By dissecting the molecular signatures of individual cells, single-cell approaches provide insights into the mechanisms driving persistent inflammation, impaired angiogenesis, hormonal dysregulation, and immune dysfunction in endometriosis. Importantly, emerging evidence indicates that infertility and reduced assisted reproductive technology (ART) success in endometriosis reflect coordinated cellular and molecular dysfunction rather than solely anatomical abnormalities. Single-cell analyses of oocytes, granulosa cells, and endometrial cell populations demonstrate transcriptomic and epigenetic alterations affecting mitochondrial function, steroid metabolism, immune regulation, and implantation-related signaling pathways, offering a biological explanation for impaired implantation and variable ART outcomes. Integration of these findings with clinical observations supports the concept that endometriosis-associated reproductive failure arises from combined ovarian and endometrial defects detectable at the cellular level. Current single-cell studies highlight candidate biomarker signatures with the potential to improve patient stratification, predict ART outcomes, and guide individualized therapeutic strategies. As these discoveries are refined into clinically applicable biomarker panels, single-cell technologies are poised to bridge mechanistic understanding and precision reproductive medicine, enabling more personalized management approaches aimed at restoring reproductive competence in patients with endometriosis.

## 1. Introduction

Endometriosis is a chronic estrogen-dependent disease in which endometrial epithelium and stroma grow outside the uterus causing local inflammation and chronic pain. Moreover, pelvic endometriosis is associated with reduced fertility. Pelvic endometriosis can be classified into three major types depending on lesion location: peritoneal, ovarian and deep infiltrating endometriosis [1,2]. The pathophysiology of endometriosis is multifactorial, encompassing genetic, hormonal, immune, and environmental factors. The most acceptable theories on the pathogenesis of endometriosis include the concepts of retrograde menstruation, vascular or lymphatic dissemination, and coelomic metaplasia [3]. It has been estimated that endometriosis affects nearly 190 million women globally, with wide disease heterogeneity [4,5,6]. Approximately 10% of women experience symptomatic endometriosis with an annual incidence of 2 to 7 per 1000, while another 11% of cases may go undiagnosed [7,8,9]. Intriguingly, evidence exists, suggesting that transient endometriotic lesions may occur in most women [10,11].

Τhe revised American Society for Reproductive Medicine (rASRM) classification defines four stages of endometriosis, ranging from minimal superficial lesions (Stage I) to extensive deep infiltrating lesions with adhesions and ovarian endometriomas (Stage IV) [12]. It is noteworthy that severity of symptoms does not always correlate with disease stage. At the cellular and molecular level, lesion persistence and progression are driven by enhanced adhesion, tissue invasion, cell proliferation and dissemination, and resistance of endometrial stem cells to apoptosis, including endometrial mesenchymal stem cells (eMSCs) and endometrial epithelial progenitors (eEPs) [13,14]. Other mechanisms leading to maintenance and progression of endometriosis include heterogeneity introduced by endometrial stromal cells (ESCs) and smooth muscle cells (SMCs), hormone resistance, and the activation of key signaling pathways such as PI3K/Akt, Wnt/β-catenin, and Janus kinase/signal transducers and activators of transcription (JAK/STAT). Furthermore, chronic inflammation mediated by cytokines sustains immune evasion, angiogenesis, and ultimately compromises reproductive outcomes. Further evidence indicates that complex interactions among genetic, epigenetic, hormonal, and environmental factors drive variable disease progression and diverse phenotypes that cannot be fully explained by a single etiopathogenetic model [15,16,17,18,19].

Despite the first successful in vitro fertilization (IVF) over 45 years ago [20] and the subsequent introduction of numerous clinical and laboratory ART procedures, overall success rates remain modest. Regional registry data from the US, Canada, the UK, Australia/New Zealand, Latin America, and Japan show a decline in live birth rates per ART cycle from 30% in 2010 to 22% in 2016, with preliminary evidence suggesting that the trend continues [21]. In women with endometriosis, ovarian response is affected in cases of endometriomas larger than 4 cm, while follicular steroidogenesis remains normal. However, endometriosis may still impair ART outcomes via altered embryo development and endometrial receptivity [7,21]. Recent studies have explored molecular mechanisms underlying gamete development and maturation, fertilization, early embryonic growth, implantation, endometrial receptivity, and the outcomes of ART techniques [22] and growing consensus highlights the importance of personalized, molecularly guided approaches over routine standardized protocols [23,24,25]. Endometriosis further complicates these challenges, as it remains one of the leading causes of infertility among women of reproductive age, affecting up to about half of infertile patients and increasing the risk of infertility by two to fourfold [26,27]. As mentioned above, the impact of endometriosis on infertility spans across multiple stages of reproduction, impairing oocyte competence, embryo quality, and endometrial receptivity through inflammatory signaling, hormonal dysregulation, and immune alterations.

Advances in single-cell technologies have begun to unravel the cellular heterogeneity within endometriotic lesions and eutopic endometrium, revealing critical molecular pathways that shape disease behavior and reproductive potential. These findings underscore the importance of integrating high-resolution cellular profiling into clinical practice in order to refine diagnostic tools and optimize personalized ART interventions. These approaches also enable the identification of reliable ovarian and endometrial biomarkers, which may serve as measurable indicators of physiological and/or pathological states and may guide early detection, treatment monitoring, and prediction of ART outcomes [28].

In the present review article, we use the term “endometriosis lesions” to refer to ectopic endometrial tissue found outside the uterus, including peritoneal, ovarian, and deep-infiltrating lesions. In this context, the present review aims to synthesize emerging single-cell evidence through a focused conceptual framework addressing three central questions:

(1) Do certain cellular populations and transcriptional programs identified by single-cell analyses contribute to endometriosis persistence and endometriosis-associated infertility?
(2) Do molecular alterations revealed at single-cell resolution represent driving mechanisms of disease or do they merely represent secondary or reactive changes?
(3) How did single-cell studies change current models of endometriosis pathogenesis and what are the implications of these insights for ART outcomes and personalized therapeutic strategies?

To ensure comprehensive coverage of the literature, the following search strategy was applied. We conducted a structured search in PubMed, Scopus, and Mendeley. The search included studies published over the past five years (September 2021–September 2025). Keywords used were “endometriosis,” “single-cell,” “assisted reproductive technology,” and “in vitro fertilization,” with appropriate Boolean operators to capture relevant combinations. Additional terms such as “oocyte quality,” “endometrial receptivity,” “granulosa cells,” and “transcriptomics” were included to capture mechanistic and translational studies. We included original research articles, reviews, and preclinical studies in humans and relevant animal models. Only articles in English were considered. Reference lists of included papers were also screened to identify additional studies. Titles and abstracts were independently screened for relevance, and full texts were reviewed to extract key findings related to single-cell analyses, endometriosis pathophysiology, and ART outcomes.

## 2. Single-Cell Insights into Endometriosis

Recent single-cell transcriptomic studies have mapped normal, eutopic endometrium and endometriosis lesions, revealing key disease-specific changes. The tissue microenvironment, including immune cells, seems to play a critical role in both normal endometrial development and progression of endometriosis [29,30,31,32]. Early transcriptomic analyses initially relied on bulk RNA sequencing to assess cellular RNA and characterize tissues under physiological and pathological conditions, providing average gene expression values from heterogeneous samples and supporting biomarker identification, prognosis, and gene fusion discovery [33]. On the other hand, single-cell RNA sequencing (scRNA-seq) enables gene expression profiling at single-cell resolution, revealing distinct cell states and cellular heterogeneity [34,35]. Advances in scRNA-seq and organoid culture systems allow detailed investigation of the complex cellular interactions and heterogeneity within the endometriosis lesions and eutopic endometrium [36].

Single-cell RNA sequencing (scRNA-seq) remains the core approach for characterizing lesion-specific cell states and rare subpopulations across the eutopic endometrium, the peritoneum, and ovarian tissues. This technology enables the generation of comprehensive atlases that link epithelial and stromal programs to disease remodeling and infertility, thus facilitating the discovery of previously unrecognized cell types, providing detailed insights into cellular differentiation paths and uncovering intricate gene regulatory networks [37,38,39,40,41,42]. When applied to endometriosis, single-cell approaches have revealed significant cellular heterogeneity, particularly in relation to fibrosis and angiogenic processes [43] and have uncovered distinct cell subtypes alongside immune dysregulation within the peritoneal environment [44]. Integration of single-cell data with large-scale transcriptomic analyses has thus provided comprehensive molecular insights into disease pathogenesis and identified potential therapeutic targets [45,46].

Taken together, findings across independent datasets consistently demonstrate marked cellular heterogeneity involving stromal, epithelial, and immune compartments; however, variability in reported transcriptional signatures likely reflects differences in tissue origin, lesion subtype, and patient cohorts analyzed. Interpretation of these results should therefore consider methodological factors inherent to single-cell approaches, including tissue dissociation effects and differential representation of fragile or rare cell populations, which may influence detected cellular states.

Rather than representing isolated observations, single-cell studies collectively converge on a limited number of recurring biological mechanisms underlying endometriosis pathophysiology. Across independent datasets, three interconnected processes consistently emerge: (i) stromal cell reprogramming and impaired decidualization, (ii) immune dysregulation within the eutopic endometrium and endometriosis lesions, and (iii) altered epithelial differentiation associated with reduced endometrial receptivity. These coordinated alterations reshape tissue homeostasis and provide a mechanistic framework linking cellular heterogeneity to lesion persistence and infertility. Importantly, several of these mechanisms directly influence implantation competence and may therefore contribute to reduced ART success.

### 2.1. Stromal Cell Reprogramming and Progesterone Resistance

A central and reproducible finding across scRNA-seq datasets is extensive stromal remodeling associated with impaired decidualization and progesterone resistance. Endometriosis affects pelvic organs as superficial peritoneal endometriosis (PEM), ovarian endometriosis (OEM) or deep-infiltrating endometriosis (DIE), lesions penetrating >5 mm. Proposed origins include retrograde menstruation, Müllerian remnant metaplasia, or ovarian surface invagination [47]. Konrad et al. found no evidence supporting the metaplasia theory in patients with Mayer–Rokitansky–Küster–Hauser (MRKH) syndrome [48].

DIE lesions exhibit enhanced fibrosis, epithelial–mesenchymal transition (EMT), fibroblast–myofibroblast transition (FMT), and smooth muscle metaplasia (SMM) [49]. Single-cell RNA sequencing identified five major cell types and 44 subpopulations, highlighting subtype-specific EMT differences and FN1–PI3K–AKT–mediated mesothelial–stromal crosstalk implicated in progesterone resistance [50].

Differential activation of the AKT pathway across lesion subtypes further supports stromal dysregulation as a disease-defining feature. Analysis of 55,000 single cells identified multiple fibroblast populations with distinct inflammatory, angiogenic, and extracellular matrix remodeling signatures enriched in MAPK, TNF, IL-17, and TGF-β pathways [51]. Fibroblasts from eutopic endometrium displayed transitional characteristics toward endometrioma phenotypes involving TGF-β, MAPK, Rho-ROCK, NF- κB, JAK/STAT, and EMT signaling. Taken together, these observations support a developmental continuum between eutopic endometrial and endometriotic stromal states, suggesting that stromal reprogramming represents a primary biological mechanism rather than a secondary inflammatory consequence [52].

### 2.2. Immune Dysregulation and Inflammatory Microenvironment

Immune imbalance represents a second major mechanism consistently identified by single-cell analyses. Large-scale atlases involving >370,000 cells demonstrated tissue-specific remodeling characterized by pro-inflammatory activation, complement upregulation, and ARID1A-associated angiogenic and lymphangiogenic programs with lymphatic endothelial expansion [37]. Complementary profiling integrating organoids and imaging mass cytometry identified an immunotolerant peritoneal niche and a perivascular mural cell population promoting angiogenesis and immune trafficking [36].

Single-cell analysis of eutopic endometrium identified altered immune composition, including reduced cyclical variation in uterine NK cells and T cells, decreased IL-10, increased pro-inflammatory cytokines, and enhanced immune–epithelial signaling during the implantation window [53]. Expanded immune profiling further demonstrated complex inflammatory communication networks involving monocytes, NK cells, fibroblasts, and epithelial cells [54].

Analysis of menstrual efflux confirmed decreased uNK populations alongside increased B cells and pro-inflammatory stromal fibroblasts exhibiting impaired decidualization, consistent with progesterone resistance [55,56,57]. These immune alterations may impair clearance of senescent cells and disrupt embryo implantation, thereby linking inflammatory dysregulation with reproductive failure.

### 2.3. Altered Epithelial Differentiation and Endometrial Receptivity

Alterations in epithelial differentiation constitute a third recurring biological theme with direct reproductive implications. Epithelial subtypes display abnormal expression of CXCL14, PAEP, CCL20, MMP7, and SFRP4, alongside enrichment in migration and motility pathways [54]. Cell-communication analyses revealed extensive inflammatory signaling between epithelial and immune compartments, suggesting disrupted epithelial–immune coordination.

**Figure 1:**
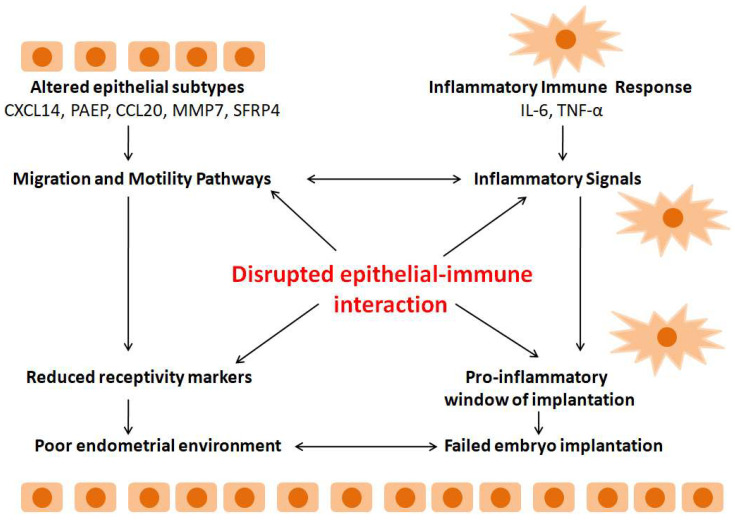
Disrupted Epithelial–Immune Crosstalk and Impaired Endometrial Receptivity in Endometriosis.

Across independent studies, reduced epithelial receptivity markers and a pro-inflammatory implantation window consistently emerge, indicating an adverse environment for embryo implantation and pregnancy establishment [53,54]. These findings provide a mechanistic explanation for impaired implantation observed clinically in patients with endometriosis undergoing ART. An overview of disrupted epithelial–immune crosstalk and impaired endometrial receptivity in endometriosis is presented diagrammatically in Figure 1.

### 2.4. Integration Across Lesion Subtypes and Study Designs

While large-scale atlases provide convergent evidence for angiogenic remodeling and immune adaptation, direct comparison between studies remains challenging due to differences in hormonal treatment status, sampling strategies, and cohort composition. Small sample sizes typical of single-cell studies may limit generalizability, emphasizing the importance of integrating findings across datasets. Despite methodological variability, single-cell investigations consistently converge on coordinated alterations across stromal, immune, and epithelial compartments. The most robust emerging insight is that infertility associated with endometriosis likely arises from multicellular network dysfunction rather than abnormalities confined to a single cell type, providing a unifying framework linking cellular heterogeneity to disease persistence and reproductive impairment.

## 3. Immune Dysregulation and Inflammatory Signaling in Endometriosis

Endometriosis is characterized by the growth of endometrial-like tissue as lesions outside the uterus, commonly on the peritoneum and/or the ovaries, causing a persistent local inflammatory condition and immune dysregulation. Immune cells are classified into the lymphoid and myeloid lineages. The lymphoid lineage includes T cells and B cells and proliferative cells specifically show high expression of DNA topoisomerase II alpha (TOP2A) and the marker of proliferation Ki-67 (MKI67), indicating active proliferation. The myeloid lineage includes dendritic cells (DCs), monocytes, and macrophages. Conventional type 1 DCs (cDC1s) and LAMP3-positive DCs express indoleamine 2,3-dioxygenase 1 (IDO1), suggesting immunosuppressive activity via L-tryptophan metabolism through the kynurenine pathway (KP) [58].

Macrophages appear to play a central role in endometriosis pathophysiology, representing both a reproducible feature across studies and a potential therapeutic target. Macrophages are divided into five subsets, including MΦ-C1q expressing C1QA and C1QB, markers associated with immunosuppressive macrophages and CD163 and MRC1, characteristic of M2 cells, as well as MΦ-CD3 expressing T-cell receptors [59,60]. While the exact proportions of these subsets vary across lesions and patients, the repeated identification of immunosuppressive macrophage populations supports their key role in maintaining a pro-tolerant microenvironment. NK and CD8^+^ T cells were reduced in ovarian endometriosis, whereas plasma cells were enriched, indicating a more immunosuppressive ovarian local microenvironment. Peritoneal macrophages are key for immune surveillance and include two major subsets, with large peritoneal macrophages (LpM) being tissue-resident, abundant and mainly embryonically derived but partially replenished by monocytes in adulthood [61]; although monocyte-derived LpM acquire characteristics of embryo-derived LpM, they exhibit some transcriptional and functional differences [62,63], while small peritoneal macrophages (SpM) are fewer and continuously renewed from infiltrating monocytes and dendritic cells (DC) [61]. Inflammation disturbs this balance, reducing LpM and promoting influx of inflammatory macrophages [63]. In endometriosis, macrophages originate from eutopic endometrium, resident LpM and recruited monocytes [64].

Single-cell RNA sequencing in a mouse model revealed two major macrophage populations residing within lesions: (a) tumor-associated macrophages (Folr2, Mrc1, Gas6, Ccl8+) that promoted Col1a1 and Tgfb1 expression in human endometrial stromal cells and enhanced angiogenesis in endothelial cells, and (b) scar-associated macrophages (Mmp12, Cd9, Spp1, Trem2+), linked to fibrosis and matrix remodeling, along with a pro-resolving large peritoneal macrophage subset (Apoe, Saa3, Pid1) associated with altered lipid metabolism and cholesterol efflux; Apoe mimetic treatment reduced lesion size and fibrosis [65]. Interestingly, cross-species bioinformatic analysis revealed strong similarities and specific differences between mouse and human macrophage populations highlighting conserved therapeutic targets [65]. These mouse findings, together with cross-species comparisons, suggest conserved macrophage-driven mechanisms promoting angiogenesis and fibrosis, highlighting potential translational targets.

In another study analysis of the nuclei dataset revealed a decreased abundance of decidualized stromal cells alongside an enrichment of uterine macrophage populations (uM), with a notable increase in the uM1 subset, in endometriosis samples [38]. uM1 cells exhibited strong upregulation of inflammatory genes (TNFRSF1B, CEBPD), confirming enhanced inflammatory activity [66,67], while uM2 macrophages showed elevated insulin-like growth factor 1 (IGF1) expression, consistent with prior murine studies [68].

Immune cell analysis revealed distinct differences between normal endometrium, endometriomas, and matched eutopic tissue. In endometriomas, T-cell and uNK cell frequencies were lower, uNK cells showed increased activity, and macrophages were more abundant with tissue remodeling characteristics compared to eutopic endometrium [37,51], which led to the promotion of a proinflammatory and angiogenic environment during the proliferative phase by immune cell subtypes including fibroblasts [51]. Endometriomas exhibited immune cell and complement activation and they were enriched in B cells and plasma cells, suggesting potential infection and highlighting a unique role for B cells in endometriosis pathophysiology. Peritoneal lesion samples showed increased mast cells and T/NK-T cells interacting with new immune targets, while some histologically normal mesothelial areas unexpectedly displayed disease-related signatures, likely influenced by variability in pathologists’ assessment of lesion depth [69,70,71,72].

**Figure 2:**
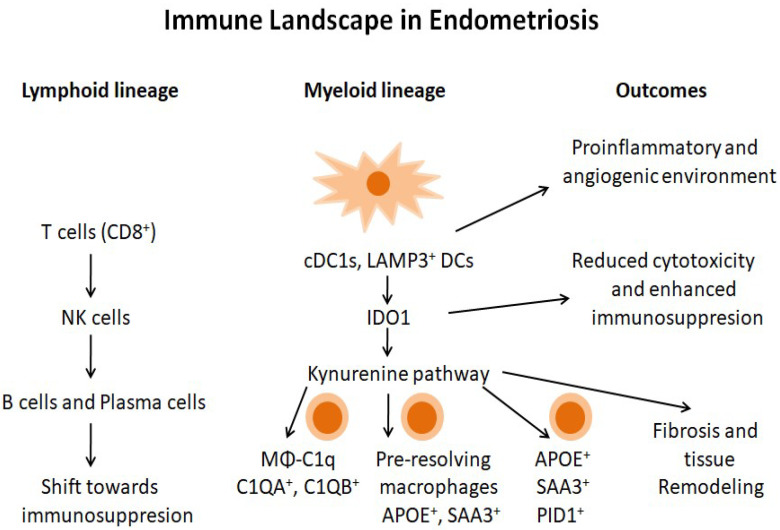
Diagrammatic overview of the immune landscape in endometriosis.

In summary, single-cell analyses consistently highlight that endometriosis is characterized by a coordinated network of immune alterations, including macrophage-driven inflammation, reduced cytotoxic lymphocytes, increased immunosuppressive dendritic cells, and enrichment of plasma and B cells in specific lesion sites. These immune changes interact with stromal and epithelial compartments, promoting fibrosis, angiogenesis, and a local pro-inflammatory environment that likely contributes to impaired endometrial receptivity and infertility. While specific immune cell proportions and activation states vary across patients, lesion types, and tissue processing protocols, the recurring patterns of immune dysregulation across independent studies suggest these features represent robust hallmarks of endometriosis pathophysiology rather than isolated observations. Taken together, this evidence supports the notion that targeted modulation of macrophage function, immune tolerance pathways, and lymphoid–myeloid interactions could represent promising therapeutic avenues, while emphasizing the need to account for inter-patient variability and methodological limitations when interpreting single-cell datasets. An overview of the immune landscape in endometriosis is presented diagrammatically in Figure 2.

## 4. Altered Stromal and Epithelial Cell Functions in Endometriosis

Endometriosis is diagnosed histologically by the presence of endometrial-like epithelial and stromal cells outside the uterus. Recent scRNA-Seq analyses demonstrated the presence of progenitor-like epithelial populations [36] and distinct transcriptional profiles across lesion subtypes, emphasizing cellular plasticity in endometriosis [37]. The Human Endometrial Cell Atlas (HECA) was essential for providing cellular context to a large-scale endometriosis Genome-Wide Association Study (GWAS) [73], enabling the identification of two decidualized stromal cell subtypes, dStromal early and dStromal mid, as endometriosis-relevant [38]. In the stromal compartment of affected cases, changes in gene expression indicated alterations in Wnt signaling (WNT) and insulin signaling pathways. Growth regulation by estrogen in breast cancer 1 (GREB1), a GWAS-linked gene induced by WNT signaling [74,75], was significantly upregulated, whereas the WNT inhibitor, dickkopf WNT signaling pathway inhibitor 1 (DKK1) was downregulated in both endometrial stromal subtypes, suggesting sustained WNT activity in the secretory-phase endometrium. Similarly, IGF1, another GWAS-associated gene, was markedly upregulated and IGF2 downregulated in these cells, indicating that proliferation and differentiation processes are likely disrupted in endometriosis [76].

Using single-cell tagged reverse transcription sequencing (STRT-seq), Yan et al. [77] analyzed samples from 11 healthy women and 23 patients with ovarian endometriosis, and identified an expansion of ciliated epithelial cells with reduced estrogen sulfotransferase (SULT1E1), an enzyme that inactivates estradiol via sulfation [78]. This likely increases estradiol, promoting proliferation and blocking apoptosis through mitogen-activated protein kinase (MAPK) signaling and B-cell lymphoma 2 (BCL2) [79]. Eutopic cells showed BCL2 and MAPK-related gene (MEF2C, RAF1) upregulation, while endometriotic lesions enhanced survival via nicotinamide N-methyltransferase (NNMT) regulation of forkhead box protein O1 (FOXO1)–BCL2-interacting mediator of cell death (BIM) signaling and sustained chronic inflammation through cluster of differentiation CD4^+^ T cells and human leukocyte antigen (HLA) class II–interferon gamma (IFN-γ) feedback [80], leading to T-cell exhaustion [81]. Immunohistochemistry (IHC) confirmed increased ciliary markers forkhead box protein J1 (FOXJ1) and tubulin polymerization-promoting protein family member 3 (TPPP3) [82,83], co-expressed with acetylated α-tubulin, in eutopic compared with normal endometrium. Compared to normal stroma, eutopic stromal cells exhibit substantial transcriptional changes that activate MAPK and insulin signaling pathways, thereby enhancing IGF-driven proliferation and survival mechanisms, which may facilitate their persistence at endometriotic lesions [77].

Another study [50] showed that two epithelial subpopulations, epithelial cells expressing collagen type I alpha 1 chain (Epi-COL1A1) and epithelial cells expressing alpha-smooth muscle actin 2 (Epi-ACTA2), were identified as non-classical epithelial cells expressing fibroblast-associated genes such as collagen type I alpha 1 chain (COL1A1) and alpha-smooth muscle actin 2 (ACTA2), exhibiting epithelial-to-mesenchymal transition (EMT) features, and associated with angiogenesis. Both subpopulations showed high expression of zinc finger E-box-binding homeobox 2 (ZEB2), and the mesenchymal marker fibronectin 1 (FN1) was highly expressed in these cells as well as in mesothelial cells, with Epi-COL1A1 particularly enriched in ovarian endometrioma lesions. Stromal cells in the same study were classified into fibro C7 cells expressing complement C7, perivascular cells expressing regulator of G-protein signaling 5 (RGS5), myosin heavy chain 11 (MYH11), six transmembrane epithelial antigen of the prostate 4 (STEAP4), and Ral GTPase activating protein like (RERGL), and other endometrial fibroblasts comprising non-decidualized endometrial stromal cells (eS) and decidualized endometrial stromal cells (dS), both showing high expression of hormone-associated genes including estrogen receptor 1 (ESR1), progesterone receptor (PGR), and progesterone receptor membrane component 1 (PGRMC1) [50].

dS were further distinguished by the expression of homeobox A10 (HOXA10) and homeobox A11 (HOXA11), members of the HOXA gene family that regulate Müllerian duct development, with HOXA10 and HOXA11 corresponding to the uterine and oviductal segments, respectively [84]. The presence of dS indicates that lesions may originate from the endometrium or endosalpin, supporting Sampson’s theory of retrograde menstruation.

Epithelial stem cells seem to contribute to endometriosis pathogenesis as bulk transcriptomic analysis of superficial peritoneal lesions showed enrichment of SOX9^+^LGR5^+^ markers in the proliferative phase compared to normal endometrium (*n* = 42) and peritoneum (*n* = 12) [31]. This enrichment, reflecting P4-resistance [56,85], indicates that the expansion of SOX9^+^LGR5^+^ stem-like cells drives endometriotic lesion development from dysfunctional basalis-derived epithelium [31,72].

In summary, single-cell analyses reveal that endometriotic lesions are maintained through coordinated alterations in stromal and epithelial compartments, including sustained WNT and IGF signaling, expansion of progenitor-like epithelial populations, and epithelial-to-mesenchymal transition. These features appear consistently across multiple cohorts and lesion types, suggesting that cellular plasticity, hormone resistance, and survival signaling are central drivers of lesion persistence rather than secondary or reactive phenomena. Importantly, these findings provide mechanistic links between molecular dysregulation and key pathological processes, including aberrant proliferation, angiogenesis, and impaired decidualization, which likely contribute to infertility in affected patients. Interpretation should consider variability due to tissue origin, lesion subtype, menstrual cycle phase, and sequencing methodology, yet the convergence of these patterns highlights robust molecular targets for future therapeutic interventions.

## 5. Intercellular Communication and Regulatory Networks in Endometriosis

Endometriotic lesions, along with the mesothelial lining, come into direct contact with the immune cells of the peritoneal fluid. Advanced analytical techniques, including mass cytometry and single-cell RNA sequencing, have demonstrated that the immune cell composition of peritoneal fluid differs markedly between individuals with endometriosis and healthy controls [86]. Zou et al. documented the emergence of two macrophage phenotypes not previously described in the context of endometriosis, detecting TCR-bearing macrophages alongside a proliferative macrophage subset in peritoneal fluid [44].

A recent study generated the most extensive single-cell atlas of endometrial tissue to date, profiling 466,371 cells from 35 endometriosis patients and 25 controls across different menstrual phases without exogenous hormone treatment [87]. Five principal cell populations were identified: mesenchymal (88.3%), epithelial (5.6%), endothelial (3.4%), lymphoid (0.9%), and myeloid (1.8%). Quantitative analysis of intercellular communication revealed increased ligand–receptor signaling in endometrium, notably through TGFβ, IL1, TNF, ICAM, VEGF, and WNT pathways, indicative of enhanced inflammation, angiogenesis, proliferation, and survival mechanisms [87].

In endometriosis, macrophages show enhanced interactions with stromal, epithelial, endothelial, and immune cells via TNF–TNFR1/2, ICAM1/2, and CXCL2/3/8–ACKR1 signaling, activating NFκB and MAPK pathways and promoting pro-inflammatory responses, leukocyte recruitment, and tissue crosstalk. These recurrent macrophage–stromal/epithelial interactions appear to be a central feature of endometriosis lesions, providing a mechanistic link between immune dysregulation and tissue remodeling. Furthermore, in endometriosis mesenchymal cells show upregulation of genes linked to focal adhesion and cytoskeletal remodelling, including ACTB, RHOA, COL4A2, COL5A2, JAK1, and MYH9, consistent with stromal-driven invasion, along with increased TGFB1 expression supporting adhesion and migration [38,88,89,90,91].

Endometriotic epithelial and mesenchymal cells exhibit increased expression of proliferation and survival genes, including NFκB and ALK pathways, alongside reduced cell death receptor signaling. Ligand–receptor analysis indicates decreased apoptosis via diminished TMEM219–IGFBP3 interactions in mesenchymal cells [88,92,93].

EGFA–VEGFR2 signaling and other angiogenesis-related ligand–receptor interactions are elevated. Mural cells, including perivascular and vascular smooth muscle cells, show increased crosstalk with endothelial cells via PGF–VEGFR1, supporting enhanced endothelial proliferation, consistent with the higher abundance of secretory mural cells in endometriosis [73,94]. These interactions underscore the importance of vascular and mural cell contributions to endometriosis lesion maintenance and angiogenesis, highlighting conserved pathways that could serve as potential therapeutic targets.

Pain in endometriosis is thought to result from interactions between endometriotic lesions and nerve fibers, driven by immune- and mesenchymal-derived inflammation that promotes sensory fiber infiltration. Endometriotic mesenchymal cells show enrichment in axon guidance and nervous system development pathways, including upregulation of CXCL12, which may enhance nerve fibre growth via the SDF-1α/Rho/mDia pathway [95,96,97,98]. Additionally, subtle increases in WNT (WNT2/3/7A–FZD3/4/6+LRP6) and NOTCH signaling were observed in endometriosis, with their interplay potentially promoting epithelial differentiation and ciliary features characteristic of endometriotic lesion [31,77]. WNT5A has been implicated in the development of endometriotic lesions through its capacity to engage multiple non-canonical WNT signaling cascades via interactions with receptors of the Frizzled and Ror families. Dysregulated WNT5A activity has been associated with the modulation of angiogenesis, metastatic progression, and inflammatory processes. Evidence further suggests that WNT5A governs collective cell migration during sprouting angiogenesis by reinforcing adherent junctions and coordinating cellular polarity. Such influences on migratory behavior and junctional stability are thought to facilitate the anchoring of cells to the ovarian stroma, thereby promoting the establishment of endometriotic lesions [39]. Accordingly, the development of targeted, non-hormonal therapeutic approaches informed by molecular insights, including the WNT5A pathway, may represent a complementary strategy to surgical management and could contribute to improved long-term outcomes in endometriosis. Future studies should explore approaches capable of modulating WNT5A signaling within endometriosis lesions while carefully evaluating potential systemic effects [39].

Experimental evidence from both in vitro and in vivo studies indicates that the peritoneal microenvironment harbors numerous small extracellular vesicles (sEVs), which may transport key mediators of endometriosis pathogenesis. Proteomic profiling of peritoneal fluid-derived sEVs identified a distinct signature in affected women, including five proteins—peroxiredoxin-1, histone H2A type-2-C, annexin A2, inter-α-trypsin inhibitor heavy chain H4, and tubulin alpha-chain—exclusively present in endometriosis patients. Among these, annexin A2 is highly expressed in stromal cell-derived sEVs from endometriosis lesions and has been shown to drive angiogenesis as well as stromal cell proliferation and migration through activation of the ERK1/2 and STAT3 pathways [99,100]. sEVs derived from endometrial stromal cells appear to enhance the migratory behaviour of normal stromal cells, drive angiogenesis, and stimulate ovarian cells to upregulate pro-inflammatory cytokines [101].

Current evidence regarding the diagnostic performance of extracellular vesicles in endometriosis remains scarce. A study reported that vesicular VEGF demonstrated an approximate sensitivity of 81% and specificity of 71% as a potential biomarker of the disease [102]. In contrast, another study, explored EV-associated microRNAs as diagnostic indicators, yielded inconclusive outcomes, largely attributable to the restricted cohort size [103]. This highlights that while EVs show promise as biomarkers, further validation in larger, standardized cohorts is required before clinical application.

Overall, single-cell and ligand–receptor analyses indicate that endometriotic lesions are orchestrated by complex intercellular communication networks, involving immune, mesenchymal, epithelial, and mural cells. Enhanced signaling through inflammatory, angiogenic, and survival pathways is a reproducible feature across independent studies, linking immune dysregulation, stromal remodeling, and lesion persistence. Mechanisms such as WNT/NOTCH crosstalk, CXCL12-mediated nerve fiber recruitment, and sEV-mediated signaling highlight multifaceted regulation of lesion establishment and maintenance. While technical and cohort variability can affect the precise magnitude of these interactions, the convergence of these pathways underscores robust molecular targets for possible future diagnostics and therapeutic interventions.

## 6. Single-Cell Biomarkers for Predicting ART Outcomes in Infertile Women Without and Women with Endometriosis

Thus far, the epigenetic implications of ART have been examined predominantly through analyses restricted to isolated genomic epigenetic layers. In several instances, studies have concentrated on targeted candidate gene regions employing array-based platforms or PCR methodologies; however, such approaches are liable to underestimate alterations that extend across the entire genome. Emerging integrative single-cell strategies provide a comprehensive framework to capture epigenetic and transcriptional variability, critical for understanding differential ART outcomes in endometriosis patients [104].

Recent advances in high-throughput technologies combined with single-cell analytical methods have given rise to multi-omic strategies that significantly extend the possibilities for investigating developmental epigenetics and genomic regulation. By integrating data from distinct epigenetic dimensions, these approaches generate an in-depth portrayal of the epigenetic variability that characterizes each embryonic cell. This integrative perspective has facilitated innovative ways of mapping regulatory sequences and clarifying their functional interplay during the extensive genomic reorganization that accompanies the earliest phases of human embryonic development [105,106,107]. By connecting epigenetic landscapes to oocyte and embryo competence, these strategies offer mechanistic insights that could guide the development of biomarkers predicting and improving ART outcomes.

Multi-omic methodologies have the capacity to uncover previously unrecognized aspects of epigenetic modification in preimplantation embryos. Beyond advancing fundamental understanding, they also provide a valuable framework for assessing the reliability of existing IVF practices and hold promises for their future optimization [108].

Initial understanding of chromosomal irregularities in early human embryonic development was achieved using fluorescent in situ hybridization (FISH). This technique, applied within preimplantation genetic testing, enabled the detection of aneuploidy as well as structural chromosomal rearrangements in single interphase blastomeres derived from cleavage-stage embryos produced via IVF [109].

Although some mechanistic insights come from animal models, these data provide a valuable reference for human ART. Using single-cell RNA sequencing (scRNA-seq), studies in pigs and mice have elucidated critical aspects of early embryo and placental development [110,111,112]. These findings highlight conserved transcriptional programs and epigenetic regulation, supporting translational relevance despite species differences. For instance, early pig embryos from IVF and parthenogenetic activation exhibit distinct mRNA decay and activation patterns, enriched in RNA processing, mitochondrial activity, DNA/H3K4 methylation, and transcription factor pathways [110,111]. In mouse placentas, frozen-thawed embryo transfer (FET) altered fetal weight and placental efficiency, with DEGs in syncytiotrophoblasts (SynTs), sinusoidal trophoblast giant cells (S-TGCs), and glycogen trophoblasts (GlyTs) affecting vascular development, oxidative stress responses, and mesenchyme formation [112]. Together, these studies provide a foundation for understanding molecular determinants of embryo competence relevant to ART.

A human study employed single-cell methylome and transcriptome sequencing (scM& T-seq) to profile DNA methylation and mRNA expression in MII oocytes and early embryos at single-cell resolution [113]. Integrated analyses revealed critical transcriptional and epigenetic milestones, including DUXA/DUXB activation and LINE-1 retrotransposon dynamics, which are essential for successful preimplantation development. Disruption of these processes may impair ART outcomes [113].

Regarding endometrial transformation across the human menstrual cycle, single-cell transcriptomics was performed on biopsies from 19 healthy ovum donors (days 4–27) [30]. Single-cell analyses in patients undergoing IVF further demonstrate the clinical relevance of these molecular insights. In 102 patients (111 cycles) with preimplantation genetic testing for aneuploidy, 412 blastocysts were profiled using single-cell DNA- and RNA-sequencing [114]. DEGs associated with trisomy 16, euploid blastocyst development, and implantation success (SHLD3, AUNIP, TFIP11, FBH1, ERCC6, OTUB2, FOXM1, and TP53) highlighted pathways involved in DNA repair, differentiation, and interaction with receptive endometrium, illustrating how single-cell profiling can informbiomarkers predicting ART outcomes.

Single-cell analyses in patients undergoing IVF further demonstrate the clinical relevance of these molecular insights. In 102 patients (111 cycles) with preimplantation genetic testing for aneuploidy, 412 blastocysts were profiled using single-cell DNA- and RNA-sequencing [114]. DEGs associated with trisomy 16, euploid blastocyst development, and implantation success (SHLD3, AUNIP, TFIP11, FBH1, ERCC6, OTUB2, FOXM1, and TP53) highlighted pathways involved in DNA repair, differentiation, and interaction with receptive endometrium, illustrating how single-cell profiling can inform predictive biomarkers for ART success.

In ovarian somatic cells, single-cell RNA sequencing revealed granulosa cell (GC) cluster differences and gene expression alterations that underlie hyporesponse to gonadotropins during IVF [115]. Although derived from a general IVF population, these findings suggest mechanisms that could similarly affect ovarian response in endometriosis, pending validation.

Finally, in endometriosis, oocyte quality is compromised even in the contralateral, unaffected ovary, indicating systemic impacts on ovarian function [116]. Ferrero et al. [117] performed single-cell RNA sequencing (scRNA-seq) on oocytes from women with ovarian endometriosis and identified 520 differentially expressed genes (DEGs) compared with healthy donors. Among these, APOE, DUSP1, G0S2, H2AFZ, ID4, MGST1, and WEE1 were upregulated, whereas PXK was downregulated. Functional enrichment analysis revealed that these genes regulate key molecular processes critical for oocyte competence, including steroid metabolism, oxidative stress response, mitochondrial activity, cell growth and cycle regulation, angiogenesis, and DNA methylation. Notably, the upregulation of stress-response and cell-cycle regulatory genes (e.g., DUSP1, WEE1) may reflect compensatory mechanisms to counteract endometriosis-associated cellular stress, whereas downregulation of PXK could impair cytoskeletal dynamics and intracellular trafficking. Overall, these transcriptomic alterations delineate a molecular landscape that may directly contribute to impaired oocyte quality and, consequently, reduced assisted reproductive technology (ART) success in endometriosis patients. This evidence underscores the importance of considering systemic ovarian effects, even in apparently unaffected tissue, when developing strategies to optimize ART outcomes in this population. Taken together, these studies demonstrate that single-cell and multi-omic approaches elucidate cellular and molecular determinants of oocyte and embryo quality, implantation potential, and endometrial receptivity, offering actionable insights for improving ART outcomes while paving the way for developing biomarkers predicting ART outcomes. An overview of these studies is presented in Table 1.

## 7. Translating Molecular Findings into Therapy and Future Perspectives

Recent research has substantially advanced our comprehension of the cellular and molecular foundations of endometriosis, highlighting mechanisms directly linked to infertility and lesion persistence, and presenting opportunities for targeted therapeutic interventions [118]. While existing treatments demonstrate clinical efficacy, emerging approaches hold the potential not only to resolve established endometriotic lesions more effectively, but also to prevent the recurrence, frequently observed after surgical management [118].

Endometriotic cells exhibit reduced apoptosis, whereas mononuclear cells in the peritoneal fluid show increased apoptotic activity, creating together a peritoneal environment conducive to disease progression. Although robust clinical data on targeted therapies remain limited, strategies promoting apoptosis in endometriotic cells represent a promising therapeutic focus [119].

Computational integrative strategies are increasingly recognized as powerful tools for enhancing both diagnostic and therapeutic approaches in endometriosis. By integrating single-cell transcriptomic data with proteomics, metabolomics, and DNA methylation—methods such as Multi-Omics Factor Analysis (MOFA) and Data Integration Analysis for Biomarker discovery using Latent cOmponents (DIABLO) can identify cell-type-specific regulatory programs disrupted in endometriosis [119,120,121,122,123]. These approaches may reveal candidate pathways for targeted therapy or biomarkers for personalized ART interventions.

Peritoneal immune cells, particularly macrophages, are highly heterogeneous and may not follow classical pro-inflammatory/pro-repair polarization. In endometriosis, macrophages show impaired phagocytic and cytotoxic functions, with increased pro-inflammatory and chemotactic activity, suggesting that modulation of these cells could serve as a therapeutic strategy [44].

Molecular targets emerging from single-cell studies include WNT5A, which is upregulated in endometriosis and enriched at engrafted endometrial junctions [39]. Modulating WNT5A may provide a non-hormonal approach to limit lesion establishment and progression, although careful evaluation of off-target effects is essential. Surgery remains critical for pain relief and fertility preservation, but small lesions are prone to recurrence; targeted therapies could complement surgical management and improve long-term outcomes.

The comprehensive mapping of the window of implantation at single-cell resolution provides a framework for precision reproductive medicine [124]. Computational tools trained on temporal endometrial atlases can assess tissue receptivity, overcoming heterogeneity challenges and enabling personalized interventions that improve ART success.

Single-cell technologies are transforming our understanding of reproductive disorders, revealing cell-type differences in endometriosis, adenomyosis, and eutopic endometrium, and highlighting niche-specific influences on disease [72]. Although single-cell RNA sequencing (scRNA-seq) is technically complex and resource-intensive, it enables the identification of key molecular signatures that can be distilled into compact biomarker panels (5–10 genes or proteins), which can subsequently be detected in non-invasive samples using PCR or ELISA, thereby facilitating their translation into routine clinical applications. These insights identify candidate diagnostic biomarkers, prognostic indicators, and therapeutic targets, supporting the development of precision medicine strategies tailored to endometriosis-associated infertility.

## 8. Conclusions

In conclusion, single-cell technologies have opened new avenues for understanding the cellular heterogeneity and molecular mechanisms underlying endometriosis, including its impact on infertility, by providing unprecedented resolution of cell types, their anatomical niches, and associated signaling pathways. Overall, single-cell studies have begun to identify specific stromal, epithelial, immune, and progenitor cell programs that may contribute to lesion persistence, immune dysregulation, and impaired reproductive function, thereby helping to clarify which cellular mechanisms are most closely linked to endometriosis-associated infertility.

These insights are beginning to elucidate disease establishment, progression, and variability among patients, offering opportunities to identify diagnostic biomarkers, prognostic indicators, and novel therapeutic targets. Importantly, high-resolution molecular profiling has enabled the distinction between core disease-associated alterations and broader inflammatory or adaptive responses, supporting a more mechanistic interpretation of hormonal, immune, and signaling abnormalities previously observed in bulk studies. At the same time, the application of single-cell RNA sequencing (scRNA-seq) has inherent limitations. Technical challenges, including biases introduced during tissue dissociation, cell capture inefficiency, and batch effects, can influence the representation of rare populations. High costs and computational complexity also restrict large-scale implementation and cross-cohort validation. Despite these challenges, scRNA-seq remains a revolutionary tool for dissecting cellular heterogeneity, uncovering rare or previously unrecognized cell types, and providing a framework for translating molecular insights into clinically relevant biomarkers and therapeutic strategies. Recognizing both its strengths and limitations ensures a balanced interpretation and guides future studies toward robust and reproducible applications in endometriosis research.

By integrating concepts of retrograde menstruation, stem/progenitor cell activity, and immune escape within a unified cellular framework, single-cell approaches refine existing models of endometriosis pathogenesis. Looking forward, combining these approaches with precisionmedicine strategies has the potential to transform the management of endometriosis-associated infertility, enabling targeted, non-hormonal interventions, improving symptom control, reducing recurrence, and ultimately advancing personalized reproductive outcomes.

## Figures and Tables

**Table 1:** Single-Cell and Multi-Omic Insights Relevant to ART and Endometriosis (including experimental models).

Cell/Compartment	Key Findings (Single-Cell/ Multi-Omic)	Biological Mechanism	ART Implications
Oocytes (endometriotic vs. healthy)	520 DEGs: APOE, DUSP1, G0S2, H2AFZ, ID4, MGST1, WEE1 (up); PXK (down) [117]	Altered steroid metabolism, oxidative stress, mitochondrial dysfunction, DNA methylation	Reduced oocyte quality; potential biomarker panel for ART success
Granulosa cells (follicular somatic)	7 GC subtypes; P4-producing GC downregulated key steroidogenic genes; ARGLU1+, SEMA3A+ clusters reduced [115]	Impaired progesterone production, altered steroidogenesis	Hyporesponsiveness to gonadotropins; predicts ovarian response and IVF outcomes
Endometrial stromal fibroblasts	Reduced decidualization markers; inflammation-driven P4 resistance [55]	Impaired decidualization, chronic inflammatory phenotype	Reduced endometrial receptivity, impaired embryo implantation
Endometrial epithelial cells	Altered expression of PAEP, GPX3, CXCL14; disrupted epithelial–immune interactions [30,54]	Impaired implantation window, pro-inflammatory milieu	Adverse embryo implantation environment; informs endometrial preparation for ART
Blastocysts (euploid vs. trisomy, human)	DEGs include SHLD3, AUNIP, TFIP11, FBH1, ERCC6, OTUB2, FOXM1, TP53 [114]	DNA repair, transcription, mitochondrial processes	Predictive transcriptomic markers for implantation potential
Embryos (single-cell methylome and transcriptome, human)	DUXA/DUXB activation, LINE-1 dynamics, DPPA2/4 timing [113]	Maternal-to-zygotic transition, epigenetic reprogramming	Milestones critical for preimplantation development; informs embryo selection
Early poig embryos (IVF and pathogenetic activation, experimental)	Highly expressed: CDV3, PCNA, CDR1, YWHAE, DNMT1, IGF2BP3, ARMC1, BTG4, UHRF2, gametocyte-specific factor 1-like [110,111]; distinct mRNA decay patterns	RNA processing, mitochondrial activity, DNA/H3K4 methylation, transcription factor pathways	Model for understanding early embryo gene regulation; informs optimization of culture conditions
Mouse placentas (FET, experimental)	DEGs in SynTs, S-TGCs, GlyTs: Igf2, Prl3b1 [112]; changes in vascular development, oxidative stress response, mesenchyme formation	Placental cell differentiation, vascular development, oxidative stress regulation	Experimental insight into FET effects on placenta; informs preclinical ART studies
Follicular fluid/bulk + scRNA integration	Altered ECM, IGF, lipid/steroid metabolism pathways; cluster composition affects ovarian sensitivity [115]	Microenvironment driven hyporesponse	Personalized prediction of ovarian response in IVF cycles

## Data Availability

No new data were created or analyzed in this study. Data sharing is not applicable to this article. Original data presented in the study are openly available in: Medline/PubMed (https://pubmed.ncbi.nlm.nih.gov/) (accessed on 1 October 2025), Scopus (https://www.elsevier.com/products/scopus) (accessed on 1 October 2025), Mendeley (https://www.mendeley.com/datasets) (accessed on 1 October 2025).

## Abbreviations

ART	assisted reproductive technology
ASRM	American Society for Reproductive Medicine
CCL20	chemokine ligand 20
DC	dendritic cells
DEGs	Differentially expressed genes
DIE	deep-infiltrating endometriosis
ECM	extracellular matrix
eMSCs	endometrial mesenchymal stem cells
EMT	epithelial–mesenchymal transition
eEPs	endometrial epithelial progenitors
ESCs	endometrial stromal cells
FISH	fluorescent in situ hybridization
FMT	fibroblast–myofibroblast transition
FN1-PI3K-AKT	Fibronectin 1–Phosphoinositide 3-kinase–Protein kinase B
GCs	Granulosa cells
GREB1	Growth regulation by estrogen in breast cancer 1
GWAS	Genome-Wide Association Study
HLA	human leukocyte antigen
IGF	Insulin-like Growth Factor
IHC	Immunohistochemistry
IL-10	interleukin-10
IVF	in vitro fertilization
JAK/STAT	Janus kinase/signal transducers and activators of transcription
MAPK	mitogen-activated protein kinase
MMP7	matrix metallopeptidase 7
MRKH	Mayer–Rokitansky–Küster–Hauser syndrome
NF-κB	nuclear factor kappa B
OEM	ovarian endometriosis
PEM	peritoneal endometriosis
Rho-ROCK	Rho-associated protein kinase
scRNA-seq	single-cell RNA sequencing
sEVs	small extracellular vesicles
SFRP4	secreted frizzled-related protein 4
SMCs	smooth muscle cells
SMM	smooth muscle metaplasia
STRT-seq	single-cell tagged reverse transcription sequencing
TGF-β	transforming growth factor beta
